# A Novel Approach of Dynamic Cross Correlation Analysis on Molecular Dynamics Simulations and Its Application to Ets1 Dimer–DNA Complex

**DOI:** 10.1371/journal.pone.0112419

**Published:** 2014-11-07

**Authors:** Kota Kasahara, Ikuo Fukuda, Haruki Nakamura

**Affiliations:** Institute for Protein Research, Osaka University, Suita, Osaka, Japan; Oak Ridge National Laboratory, United States of America

## Abstract

The dynamic cross correlation (DCC) analysis is a popular method for analyzing the trajectories of molecular dynamics (MD) simulations. However, it is difficult to detect correlative motions that appear transiently in only a part of the trajectory, such as atomic contacts between the side-chains of amino acids, which may rapidly flip. In order to capture these multi-modal behaviors of atoms, which often play essential roles, particularly at the interfaces of macromolecules, we have developed the “multi-modal DCC (mDCC)” analysis. The mDCC is an extension of the DCC and it takes advantage of a Bayesian-based pattern recognition technique. We performed MD simulations for molecular systems modeled from the (Ets1)_2_–DNA complex and analyzed their results with the mDCC method. Ets1 is an essential transcription factor for a variety of physiological processes, such as immunity and cancer development. Although many structural and biochemical studies have so far been performed, its DNA binding properties are still not well characterized. In particular, it is not straightforward to understand the molecular mechanisms how the cooperative binding of two Ets1 molecules facilitates their recognition of Stromelysin-1 gene regulatory elements. A correlation network was constructed among the essential atomic contacts, and the two major pathways by which the two Ets1 molecules communicate were identified. One is a pathway via direct protein-protein interactions and the other is that via the bound DNA intervening two recognition helices. These two pathways intersected at the particular cytosine bases (C110/C11), interacting with the H1, H2, and H3 helices. Furthermore, the mDCC analysis showed that both pathways included the transient interactions at their intermolecular interfaces of Tyr396–C11 and Ala327–Asn380 in multi-modal motions of the amino acid side chains and the nucleotide backbone. Thus, the current mDCC approach is a powerful tool to reveal these complicated behaviors and scrutinize intermolecular communications in a molecular system.

## Introduction

During the past decade, extensive efforts in the field of molecular biology have shed light on the paramount importance of non-coding regions in the human genome, which were traditionally considered as “junk”. In particular, an unexpectedly huge amount of regulatory elements have been found by recent studies [1]. Currently, the mechanistic details of gene expression regulation through these elements are not well understood, especially at the atomistic level. Since regulatory elements conduct their functions through specific binding with a certain class of proteins, *i.e.*, transcription factors (TFs), the molecular interactions between TFs and DNA are the keystone to gene expression. The DNA binding affinity of a TF is dynamically modulated by a variety of biochemical phenomena, such as post-translational modifications [2], ordering or disordering of disordered regions [3], and cooperative binding of one or more other TFs [4].

As an interesting example, the v-ets erythroblastosis virus E26 oncogene homolog 1 product (Ets1), which plays essential roles in a wide range of important biological processes, such as cancer and immunity, has been extensively studied [5]–[9]. A highly conserved region named the ETS domain consisting of a winged helix-turn-helix motif specifically recognizes the GGA(A/T) signature sequence. Many regulatory elements control the transcriptional activity of their target genes by binding with Ets1 and other partner TFs. For example, the TCRα/β gene is regulated by the cooperative binding of three TFs: the Ets1, the runt-related TF 1 (Runx1), and the core-binding factor β (CBFβ) [10]. Regulation of the mb-1 gene is established by the cooperative binding of Ets1 and paired box 5 (Pax5) to the promoter region [11]. The stromelysin-1 gene is regulated by the Ets1 homo-dimer [12]–[14]. The partner TFs are considered to affect the stability of the “inhibitory module” of Ets1, which consists of short helices upstream of the ETS domain (HI1 and HI2 helices) and downstream from it (H4 and H5 helices). Interestingly, the HI1 region adopts a helix conformation only when Ets1 is free from DNA; in other words, the formation of the inhibitory module with ordered HI1 inhibits DNA binding by Ets1 [15]. Although these phenomena have been proven by experiments, the details of the molecular mechanisms by which the partner TFs affect the Ets1–DNA binding stability are still largely unclear. Here, we have focused on the Ets1 homo-dimer with the Stromelysin-1 gene promoter [12], as an example of the cooperative binding of Ets1, to investigate the effects of an Ets1 molecule on the binding of the other Ets1 with the regulatory element at the atomistic level.

The molecular dynamics (MD) method, which simulates a time evolution of atomic coordinates based on the Newtonian mechanics, is a promising method toward illuminating the atomistic details of such complicated processes in molecular systems from the 3D structure data. It has been applied to analyze Ets1–DNA binding [16]–[18]. Reddy *et al*. analyzed binding specificity of the signature sequence with simulations of Ets1–DNA complexes with different sequences [16]. Kamberaj and van der Vaart showed that Leu337–DNA interactions works as a conformational switch of Ets1 by using the DCC analysis [17]. Karolak and van der Vaart also simulated the stability of the inhibitory module by applying the replica exchange method [18]. While the MD method provides fruitful information about atomic motions, and today’s supercomputers are able to perform long-term MD simulations of large molecular systems, extracting knowledge from a huge amount of data generated by simulations is not straightforward. For analyzing communications among separate parts of a molecular system, *e.g.*, communication among two Ets1 molecules and DNA, the dynamic cross correlation (DCC) analysis has been extensively applied to quantify the correlation coefficients of motions between atoms [19]. The DCC between the *i*th and *j*th atoms is defined by the following equation,
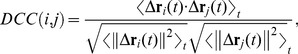
(1)where ***r*** *_i_(t)* denotes the vector of the *i*th atom’s coordinates as a function of time *t*, 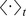 means the time ensemble average and 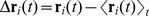. While the DCC analysis can provide insight into the correlative motions of atoms, it could overlook some kinds of correlative motions, due to its reliance on displacements from the uniquely determined average coordinate. Namely, the DCC has a definitive meaning if an atomic coordinate behaves under a uni-modal distribution. Atoms may perform multi-modal behaviors over a long time period, especially for the side-chains of amino acids, which rapidly move with flipping motions. Although the DCC has usually been applied for analyses of backbone fluctuations and domain motions by focusing on only the Cα atoms, observations of side-chain interactions are also important for our purposes. Therefore, we developed a new method to extend the conventional DCC, by explicitly including the multi-modal motions of atoms. We call the method “multi-modal DCC” (mDCC). In addition, we used techniques in the field of complex network analyses for visualization and investigation of communications among molecules, via the atomic correlative motions in a molecular assembly.

In this article, we first introduce our new method to analyze atomic correlative motions on the MD trajectory, mDCC. We then report the results of MD simulations on the crystal structure of the (Ets1)_2_–DNA complex (PDB ID: 3MFK) and three other models constructed from this crystal structure: the single Ets1–DNA complex constructed by removing an Ets1 (chain B), the model with the N380A mutation in the both Ets1 molecules, and the isolated double-stranded DNA extracted from the crystal structure. In total, 900 ns trajectory data (200 ns for each, and an additional 100 ns run of the (Ets1)_2_–DNA model with another set of initial atomic velocities) were analyzed by using the mDCC method, and the results were visualized as heatmaps, the 2D and 3D network diagrams. We discuss the communication between the two Ets1 molecules and how it affects Ets1–DNA binding.

## Results and Discussion

### Multi-modal Dynamic Cross Correlation Analysis

Here, we introduce a new method, named mDCC, that quantifies the correlation of motions between atoms moving under multi-modal distributions. In this approach, we first build a spatial distribution of atomic coordinates sampled from a MD trajectory by a Gaussian mixture distribution, which is a linear combination of Gaussian functions,
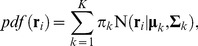
(2)

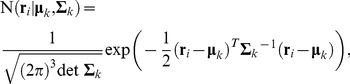
(3)where ***r*** *_i_* denotes a coordinate of the *i*th atom, *N* is a three-dimensional Gaussian function, and π*_k_* is a weighting coefficient. **µ** *_k_* and **Σ** *_k_* indicate the parameters for the *k*th Gaussian element: a 3D vector of mean coordinate, and a symmetric, positive-definite 3×3 matrix, respectively. By imposing conditions 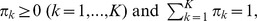 Eq. (2) represents a probability density function for the event that the *i*th atom is observed at the position ***r*** *_i_*. In contrast to the conventional DCC analysis, which uses only one averaged coordinate, our approach decomposes the atomic motions into *K* modes, or Gaussian functions, and calculates deviations from the individual *K* “averages.” These parameters π*_k_*, **µ** *_k_*, and **Σ** *_k_* were learned from a trajectory by applying a pattern recognition technique based on a variational Bayesian approach [20]. In this approach, the assignments of each data point ***r*** *_i_* to the Gaussian functions and the estimations of parameters of the Gaussian mixture distributions were iteratively updated in order to obtain the parameter values with the maximum likelihood, which is based on a variational approximation. The k-means clustering was employed with randomly generated initial assignments in order to generate an initial guess of the assignments for the variational Bayesian approach. Here, the maximum number of modes of each atom (the parameter *K*) was set to five. If an atomic motion was likely to behave under a smaller number of modes than five, then the weighted value π*_k_* of excess modes should be close to zero in the learning process (we omitted minor modes with π*_k_*<0.01).

Second, on the basis of the inferred Gaussian mixture distributions, the correlation, 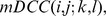 between the fluctuation of the *i*th atom from the *k*th mode (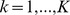) and that of the *j*th atom from the *l*th mode (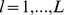) is defined by the following equation,
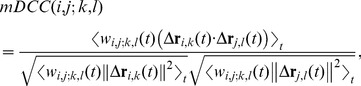
(4)

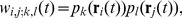
(5)

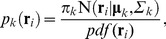
(6)where 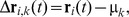 and 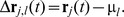 Here, *p_k_(* ***r*** *_i_)* can be considered as the degree of the assignment of the *i*th atom coordinate to the *k*th mode, for which 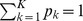 holds. Eq. (4) is different from Eq. (1) in several respects. First, it does not use just the single average 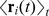, but instead uses a number of “averages,” *viz.*, several modes 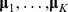, in order to distinguish atomic fluctuations from the individual mode, each of which can be viewed as a quasi-stable position. Second, Eq. (5) introduces a weight, 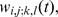 to observe the relationship between the two atom motions for which the *i*th atom is near 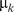 and the *j*th atom is near 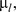 by emphasizing the specific simulation duration. Namely, 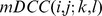 is mainly calculated from the time ranges when the *i*th and *j*th atoms simultaneously belong to modes *k* and *l*, respectively, by using the coefficient 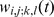 weighting these time ranges. For aiming this emphasizing effect, we use the common weight, 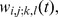 for the two terms in the denominator of Eq. (4), although it may seem to be peculiar from the viewpoint of the basic definition of a correlation coefficient. However, it should be noted that the normalized property, 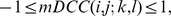 is ensured, since 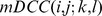 measures the cosine of the angle between two vectors in 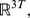 one is 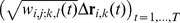 and the other is 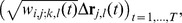 where *T* is the total number of the time ensemble. The mDCC can compensate for the weakness in the DCC analyses by detecting hidden information about the multi-modal behaviors of atomic motions.

In practice, when *K* and *L* Gaussian functions with 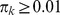 and 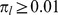 were found for the *i*th and *j*th atoms, respectively, then the *K*×*L* mDCC values were defined for this atom pair. In this paper, we mainly analyze the maximum value of mDCC for each pair of atoms or residues, and mode pairs with 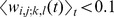 were omitted because of the very few co-occurrences of these pairs of fluctuation modes.

To illustrate the characteristic features of the mDCC and its advantages over the conventional DCC, a simple toy model consisting of two oscillating particles was analyzed by the mDCC method, and the results are shown in Fig. S1 (see also Movie S1).

### Overview of the mDCC Analysis of the (Ets1)_2_–DNA Complex

We performed the MD simulation of the (Ets1)_2_–DNA model, prepared from the crystal structure (PDB ID: 3MFK) consisting of two Ets1 molecules (chain A and B) and a double-stranded DNA. The sequences of the DNA strands are 5′-GCAGGAAGTGCTTCCT-3′ (chain C) and 5′-CAGGAAGCACTTCCTG-3′ (chain D), and we refer to each base by the residue ID defined in the PDB file, that is from G1 to T16 and from C101 to G116 for chains C and D, respectively. Then, the simulation trajectory was analyzed by using the mDCC method. As a result of a pattern recognition process for each 3D spatial distribution of 2,887 heavy atoms, 6,886 Gaussian functions (or modes) were found. Remember that the maximum number of Gaussian functions for each atom is an adjustable parameter, and five was applied in this study. Only 8.80% of heavy atoms had five Gaussian functions to approximate the distribution, and most of the atoms had smaller numbers than five (Fig. S2B). The number of Gaussian functions and the root mean square fluctuations of each atom were roughly correlated with *R^2^* = 0.383. (Fig. S2C). The atoms with the five Gaussian functions tended to be located in highly flexible regions, such as around the N-terminal HI1 helix that becomes disordered when Ets1 binds to DNA. In addition, a large part of the Gaussian functions in the atoms with the five modes were very minor (37.5% of them were 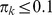 the purple part of the left-most bar in Fig. S2A).

**Figure 1:**
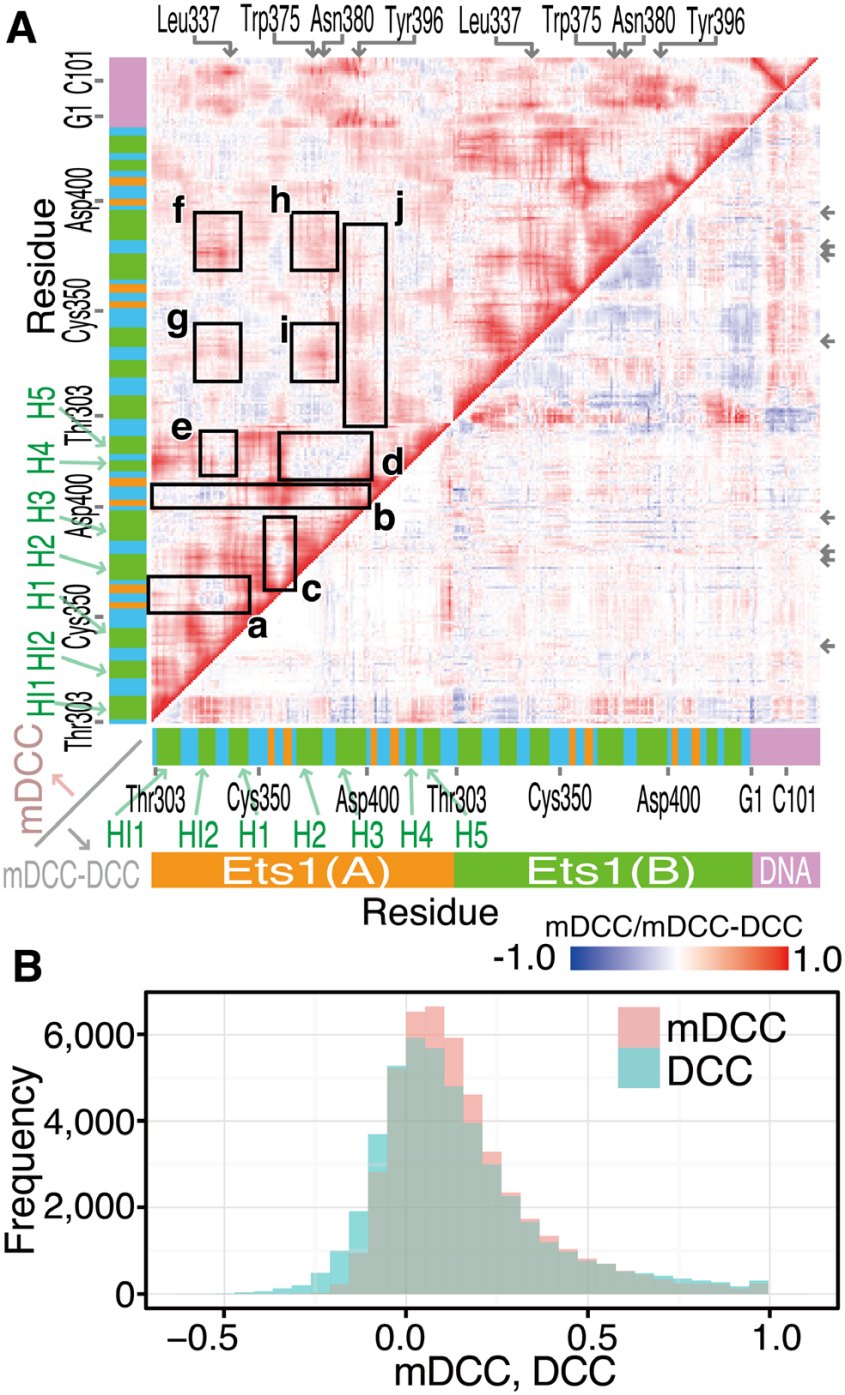
mDCC and DCC among residues of the (Ets1)_2_–DNA complex. (A) The maps of mDCC values and their differences from the DCC values (the upper and lower triangles, respectively). The color gradation from blue to red corresponds to mDCC values and their differences from DCC values from −1.0 to 1.0. The horizontal and vertical axes denote residues in the system, including two Ets1 molecules and a double-stranded DNA. The colored bars along each axis provided a guide for the secondary structures of residues: green, orange, cyan, and pink denote α-helix, β-strand, loop or turn, and DNA, respectively. Parts marked by the rectangles a–j, and residues marked at the top and right of the map are discussed in the main text. (B) Histogram of residue-wise mDCC and DCC values, shown in pink and cyan, respectively.



The mDCC map and its differences from the DCC map for all residue pairs are shown in the upper and lower triangular matrices in Fig. 1A, respectively. These values for a pair of residues *a* and *b* were defined as the maximum values in each pair of atoms with modes as follows:

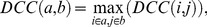
(7)

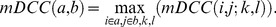
(8)


Here, to elucidate the effects of local interactions on the communications of the entire molecular assembly, we focused on highly positively correlated pairs, by taking the maximum values. Fig. 1 shows that the DCC and mDCC maps show similar features, but the mDCC values were tended to be higher than the corresponding DCC values, because the maximum values were taken from all combinations of modes in mDCC. The median DCC and mDCC values were 0.0956 and 0.114, respectively (Fig. 1B).

In order to evaluate robustness of the mDCC analysis, we calculated the mDCC maps from the following four different conditions: (i) the parameter *K* = 10, (ii) use of backbone atoms for calculating mDCC values of residue pairs, (iii) use of some different time windows in the trajectory, and (iv) another initial guess of the pattern recognition process. (i) We analyzed the trajectory with the parameter *K* = 10 in addition to the default parameter *K* = 5. Differences of the mDCC with *K* = 10 from that with *K* = 5 mainly appeared at the flexible N-terminal regions (Fig. S3A) because wide spread distributions need a large number of Gaussian functions to cover all of sampled coordinates. The Pearson correlation coefficient for mDCC values of *K* = 10 from those of *K* = 5 was 0.975. This result indicates that the mDCC values were not significantly affected by the changes of the adjustable parameter *K* value lager than *K* = 5. Thus, the value of *K* = 5 is good to represent the atomic motions. (ii) We calculated the residue pair mDCC value by taking a representative backbone atom from each residue, Cα and C5’ for amino acids and nucleotides, respectively (Fig. S3B), instead of taking the maximum mDCC value among atom pairs in each residue pair. The result shows that the overall tendencies of the mDCC map and the original one were very similar (the Pearson correlation coefficient was 0.938). Focusing on the maximum value can detect positive correlations in contacting side-chain pairs without hiding the strongly anti-correlated motions. (iii) Following the previous arguments about the convergence for the conventional DCC analysis [21], [22], the convergence of the mDCC analysis was examined by calculating the mDCC maps with the different time windows: the time range from 10 ns to 100 ns, the time range from 110 ns and 200 ns, and the time range from 10 ns to 100 ns in the alternative run with the different initial atomic velocities (Fig. S4A, B, and C). The Pearson correlation coefficients with the mDCC map calculated from 10 ns to 200 ns were 0.939, 0.923, and 0.832, respectively. The mDCC map of the alternative run was slightly different from the mDCC map of the original trajectory, where the most of differences were arisen from the motion of the inhibitory module including the disordered N-terminal region. The Pearson correlation coefficient only for the DNA-binding ETS core domain (Leu337–Phe414) was 0.910. While it is difficult to characterize the equilibrium motion of the disordered region within the 200 ns of simulation trajectory, the motions in the ETS core domain was considered to be well converged. The movies of these two trajectories are shown in Movies S2 and S3. (iv) As the pattern recognition process depends on randomly determined initial parameters, we repeated the analysis by using different random values with the same trajectory and compared the mDCC map with the original one. Consequently, the Pearson correlation coefficient of all of residue-wise mDCC values was 0.995, and the mDCC maps almost coincide with each other, except subtle differences only appearing in the flexible N-terminal region of Ets1 molecules (Fig. S4D). In summarize, while there were some differences in the mDCC values at the flexible regions among some conditions, the correlations in other regions were robust. We mainly discuss the motion of correlations among these structured regions.

**Figure 2:**
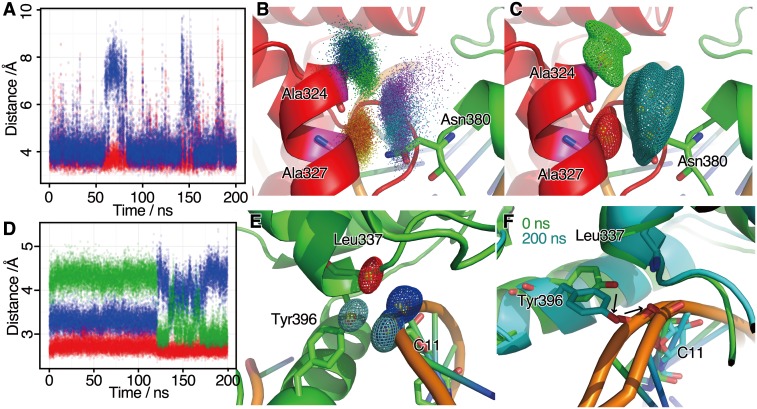
Examples of transiently formed intermolecular interactions. (A, B, and C) Interactions among the Nδ atom of the Asn380B side-chain, and the Cβ atoms of Ala324A and Ala327A. (A) The time course of interatomic distances, where red and blue plots denote Ala324A–Asn380B and Ala327A–Asn380B, respectively. (B) Spatial distributions of the coordinates of the three atoms. Color gradations of the plots, green to blue, yellow to red, and cyan to magenta, correspond to the time evolution of the simulation from 10 to 200 ns, for Ala324A, Ala327A, and Asn380B, respectively. (C) Contours of probability density functions of the Gaussian mixture models learned from the distributions in (B). The green, red and cyan meshes denote the contours for Ala324A, Ala327A, and Asn380B, respectively. (D, E, and F) Interactions among the Oη atom of Tyr396B, the backbone nitrogen atom of Leu337B, and an oxygen atom of the phosphate group of C11. (D) Time course of interatomic distances, where red, blue, and green plots denote Tyr396B–C11, Leu337B–Tyr396, and Leu337B–C11 pairs, respectively. (E) Contours of probability density functions of the Gaussian mixture models. The cyan, red, and blue meshes denote the contours of Tyr396B, Leu337B, and C11, respectively. (F) Snapshots at 0 ns (green) and 200 ns (cyan). The structures of the three residues focused on here are shown as sticks.

Comparing the DCC and mDCC values for each pair of residues revealed that there are several pairs with transiently correlated motions at the intermolecular interfaces. As examples of them, interaction of Asn380 at the two Ets1 interface and Leu337 at the interface of Ets1 and DNA are shown in Fig. 2, because these residues are known as important residues for the cooperativity by the mutation study [12]. In the first example, the side-chain of Asn380B at the H2–H3 loop interacts with Ala324A and Ala327A at the HI2 helix of the partner Ets1 (the characters A and B after the residue numbers indicate the chain ID of the Ets1 molecules). The time courses of the interatomic distances between the Nδ atom of Asn380B and the Cβ atoms of these alanine residues (Fig. 2A) and the probability density functions for each Gaussian element of the Nδ atom of Asn380B (Fig. S5) showed that Asn380B transiently flipped and switched its fluctuation modes. The spatial distributions obtained from the simulation trajectory for the atoms in Asn380B, Ala324A, and Ala327A are shown in Fig. 2B, and they were modeled as four, three, and three Gaussian functions, respectively (Fig. 2C). The mode with the highest probability in the Nδ atom of Asn380B (π*_k_* = 0.683) was highly correlated with both Ala324A and Ala327A, with mDCC values of 0.613 and 0.654, respectively. These correlations cannot be detected by using the conventional DCC method, where the DCC values were 0.282 and 0.389 for Asn380B–Ala324A and Asn380B–Ala327A, respectively. A previous experimental study showed that Asn380 and Gly333 at the partner chain are key residues for establishing intermolecular communications between the two Ets1 molecules [12]. Our simulation study showed that Asn380 transiently interacted with Ala324 and Ala327 of the partner Ets1, and further analyses imply these interactions could play an important role for the interplay of dimerized Ets1 molecules as described below.

The second example of transient interactions is the interface among the H1, H3 helices of Ets1 and DNA. The side-chain of Tyr396B in the H3 helix of the Ets1 molecule flipped, and the phosphate group of C11 of DNA slid at about 120 ns (Fig. 2F). As these two motions were coupled, the distance between these two groups did not change very much (the red plot in Fig. 2D), but the relative positions with Leu337B in H1 helix were altered. The maximum mDCC values were 0.799, 0.676, and 0.734, and the DCC values were 0.162, 0.0384, and 0.359 for the Tyr396B–C11, Leu337B–C11, and Leu337B–Tyr396B pairs, respectively. An experimental study indicated that Leu337 plays important roles in the cooperative binding of Ets1 and partner TFs and in the regulation of auto-inhibition [23]. In these examples, particular intermolecular interactions, which are crucial for molecular communications, showed multi-modal behaviors due to amino acid side-chain flipping and nucleotide backbone sliding motions. Thus, the current mDCC method is useful for finding and analyzing such transient interactions without *a priori* knowledge about mechanisms of the transitions, such as flipping, and sliding.

### Correlation Network in the (Ets1)_2_–DNA Complex

The mDCC map (the upper triangle of Fig. 1A) shows that the β-sheet region exhibited weak negative correlations with the other parts inside an Ets1 molecule (a, b and c in Fig. 1A), except for the H4 and H5 helices. These helices, which are parts of the inhibitory module, correlated weakly and negatively with the H2, H3 helices and the two loops at the interface of the protein–protein interaction (HI2–H1 and H2–H3 loops, indicated by d and e in Fig. 1A). As the intermolecular interactions, these two interface loops correlated positively with the same regions in the partner Ets1 (f, g, h, and i in Fig. 1A), because of the direct contacts. In contrast, the recognition helix H3 correlated positively with the entire region of the partner Ets1, although they did not contact directly. This correlation of the recognition helix with the partner implies that binding with the partner affects the recognition of the regulatory element by altering the motions of the recognition helix.

**Figure 3:**
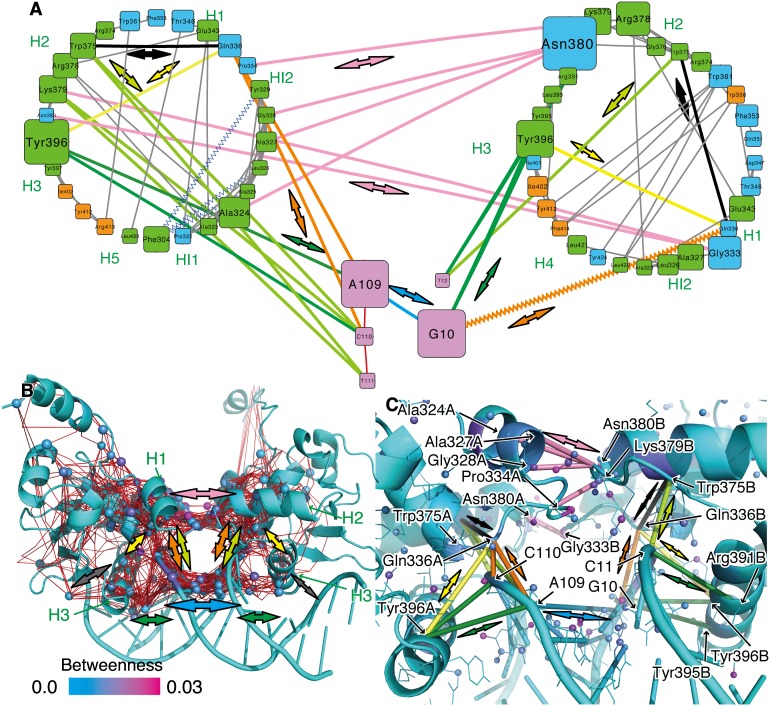
A correlation network in (Ets1)_2_–DNA model. (A) A simplified correlation network diagram in two-dimension (2D) as a sub-network of the original one shown in Fig. S7. Each node indicates a residue and each edge indicates a proximal residue pair with a highly positive correlation (the maximum value of mDCC ≥0.5 and the distance between the center positions of the modes <5.0 Å). The two circles correspond to the two Ets1 proteins (chain A and chain B correspond to the left circle and right circles, respectively), and the pink nodes are the DNA. The colors of the Ets1 nodes represent secondary structures: green, orange, and cyan indicate α-helix, β-strand, and others, respectively. The sizes of nodes denote the Betweenness values of residues. Important interactions mentioned in the manuscripts are shown as colored edges with bold arrows. (B) A 3D representation of the core network. The colors of atoms and ribbons represent their Betweenness values, and the atoms in the top 5% Betweenness are shown as spheres. Red lines indicate the shortest paths among all of the spheres. (C) The 3D structure around the recognition (H3) helix and the intermolecular interfaces. The pairs of residues corresponding to colored edges in Fig. 3A are shown as cylinders.

In order to analyze the propagation effects of local interactions toward the entire molecular assembly, especially from the recognition helix, we focused on interacting pairs with correlative motions. Fig. S7 visualizes a network of residue pairs in a positively correlative motion, where the maximum value of mDCC is equal to or larger than 0.5, and the strongly coupling pair has a distance between the centers of the modes that is shorter than 5.0 Å. In addition, because correlation values do not directly mean the importance of the interactions, we applied the “Betweenness”, a well-known measure of centrality in the field of complex network analysis, which is defined by the following equation, in order to assess the importance of each residue for the connection of the entire network:
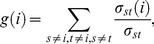
(9)where *g(i)* is the Betweenness of the *i*th node, σ*_st_* denotes the number of shortest paths between the *s*th and *t*th nodes, and σ*_st_ (i)* denotes the number of shortest paths between the *s*th and *t*th nodes via the *i*th node. For example, Betweenness becomes very high at a bridge between two cliques (Fig. S6). This value is calculated from only the topological feature of the network without direct consideration of 3D information, modes of motions, and chemical information about atoms or residues. The Betweenness values were mapped onto the network, as the colors of the nodes in Fig. S7. In order to simplify this complex network, a sub-network was created by extracting the top 20% highest Betweenness residues (Fig. 3A), where the residues without any edges are not shown. This figure summarizes the correlation networks and provides important parts of communications in the molecular system. Note that the Betweenness values were rather sensitive to the adjustable parameter *K*. The Pearson correlation coefficient of Betweenness values from the results of *K* = 10 and *K* = 5 was 0.639. However, among the top 20% of the highest Betweenness residues (61 residues) in each result of mDCC conditions (*K* = 5 and 10), 35 residues consistently appeared in the both conditions and several experimentally verified important residues and their neighbors (*e.g.*, Asn380, Pro334, Gln336, and Tyr396) were included.

Several residues around the C-terminus of the H2 helix show high Betweenness values. The residue with the highest Betweenness is Asn380, which is a main player in the intermolecular interactions between two Ets1 molecules, by forming a hydrogen bond with Gly333 and other interactions (the pink arrows in Fig. 3). In fact, their importance has been reported by mutation assays [12]. A neighboring residue, Lys379, which has the ninth and tenth highest Betweenness values for chains A and B, forms a salt bridge with the phosphate groups of T111 and T12, respectively (the lime arrows in Fig. 3). The Arg378 side-chain forms a salt bridge with the side-chain of Glu343 in the H1 helix, and it also makes a water-mediated interaction with the backbone nitrogen atom of Ala324 in the HI2 helix of the same Ets1 molecule (Figs. S8A, B, and C). The carboxyl group of Glu343 frequently flipped and changed its interacting partner nitrogen atoms in Arg378. Trp375 interacted to T111/T12 with a hydrogen bond between the nitrogen atom in the side-chain and the phosphate group (Figs. S8D, E, and the lime arrow in Fig. 3) and it also interacted to the N-terminus of the H1 helix (Ile335, Gln336, and Leu337, shown by the black arrows) with hydrophobic contacts. The second and third residues with the highest Betweenness are G10 and A109, which intervene between the two H3 helices (the cyan arrow), and they interact with Tyr396 in the H3 helix, which has the fourth and fifth highest Betweenness (the green arrows). Both Tyr396 and G10/A109 interact with Gln336 at the HI2–H1 loop (the yellow and orange arrows). On the contrary, it is interesting that the consensus sequence GGAA did not exhibit a high Betweenness, because it is located on the distal side of the DNA structure (the gray arrows in Fig. S7).

Furthermore, this network was mapped onto the 3D graphics of the complex structure with the extraction of the “core” of the network, to avoid filling up the entire structure with a massive amount of edges (Fig. 3B). The core network was defined as the top 5% highest Betweenness atoms (shown as spheres in the figure) and the shortest paths among them (red lines). The spatial positions of colored edges in Fig. 3A are indicated by the bold arrows. Details of the 3D structure around the recognition helices and intermolecular interfaces are shown in Fig. 3C, with the colored edges in Fig. 3A as the cylinders. These 3D networks visualize spatial communication pathways between the two recognition helices in the two Ets1 molecules: namely, the path through protein–protein interactions between the H2–H3 and HI2–H1 loops in each Ets1, contacting around Asn380 and Gly333 (the pink arrows in Fig. 3, corresponding to Fig. 1A f and i), and the path through DNA (the cyan arrow). While these pathways are spatially distinguishable at the middle of complexes (edges with the pink and cyan arrows in Fig. 3B), these two paths are connected in the network in each Ets1 molecule. The former pathway reaches the recognition helix via the interactions of Tyr396 with the HI2–H1 loop (Gln336) and the N-terminus of the H1 helix (Leu337 shown by the yellow arrows in Fig. 3). For the latter pathway, the bases of A109, C110, and T111 interact with the H3 helix (Arg391A, Tyr395A, Tyr396A, and Lys399A shown by the green arrows in Fig. 3). These two pathways intersect at C110/C11 via the hydrogen bonds with the Leu337 backbone (the orange arrows) and the salt bridges with Lys379 (the lime arrows).

### Cooperative Binding of the Ets1 Homo-Dimer

In order to investigate the effects of the cooperative binding of two Ets1 molecules, we built an artificial model by removing an Ets1 molecule (chain B) from the crystal structure of the (Ets1)_2_–DNA complex (PDB ID: 3MFK), and we performed a 200 ns MD simulation. This model is referred as the “single Ets1–DNA model”, hereafter. The conformation of Ets1 was not significantly changed from the native structure, with an exception at the N-terminal region including the HI1 and HI2 helices (Fig. S9). The large fluctuations of the HI1 helix during the simulation are considered to be natural, because the HI1 helix is disordered when Ets1 binds DNA and the ordered helical structure observed in the complex crystal structure is due to the crystal contacts forming a domain swapped assembly with a neighboring asymmetric unit [12].

The C-terminus of HI2 also largely fluctuated during the whole simulation, and it became partly unstructured after 150 ns in the single Ets1–DNA model, while the HI2 helix retained the initial structure during 200 ns MD for (Ets1)_2_–DNA. This is due to the dissociation of the Ala327A–Gly333A interactions caused by the loss of the intermolecular interaction between Gly333A and Asp380B (Fig. S9B, C, and D). This conformational change is an unexpected relaxation, because the loss of the partner Ets1 could facilitate the formation of the inhibitory module, by packing of the HI1, HI2, H4, and H5 helices. However, the observed behavior with large fluctuations seems to avoid the packing of these helices. This deformation can be interpreted as the first step for packing the inhibitory module. In fact, helix HI2 in the inhibitory module in an isolated Ets1 (PDB-ID: 1R36, measured by NMR [24]) is more tightly packed than that in the (Ets1)_2_–DNA complex (PDB-ID: 3MFK; Fig. S10A). Thus, repositioning of the HI2 helix could be a required to form the inhibitory module.

An additional conformational change in the inhibitory module is the formation of a head-to-tail helical dipole interaction between HI1 and H4. This phenomenon was observed during the simulation with the (Ets1)_2_–DNA model (Fig. S10B), but it did not affect the interactions between Ets1 and DNA within 200 ns of the simulation. Thus, forming the inhibitory module would require not only ordering the HI1 helix and forming its macroscopic helical dipole interaction with H4, but also repositioning the helices to more packed conformations. The observed partial deformation of HI2 may be required for the repositioning of its helical structure.

**Figure 4:**
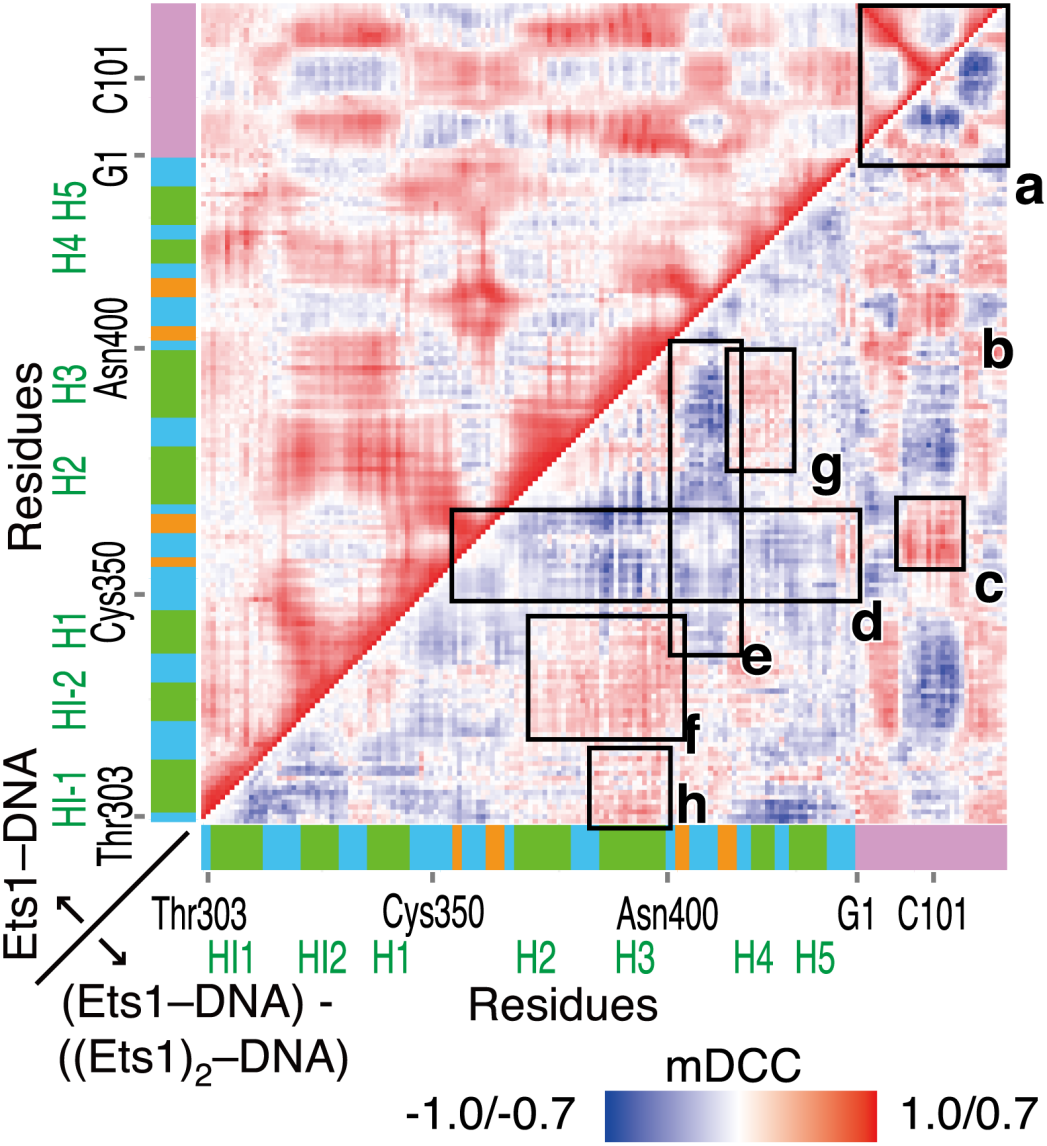
Comparisons of mDCC maps between the (Ets1)_2_–DNA and single Ets1–DNA models. The upper triangle shows mDCC values in the single Ets1–DNA model, with the color gradation from blue to red corresponding to mDCC values from −1.0 to 1.0. The lower triangle shows differences of the mDCC values in the single Ets1–DNA model from those in the (Ets1)_2_–DNA model with the color gradation corresponding from−0.7 to 0.7. For the lower triangle, negative values (blue) indicate correlations that decreased by the removal of the partner Ets1 molecule.

Next, we applied the mDCC approach to the MD trajectory of the single Ets1–DNA model and compared it with the results of the (Ets1)_2_–DNA model. Fig. 4 shows the mDCC map of the single Ets1–DNA model and its differences from the (Ets1)_2_–DNA model as the upper and lower triangles, respectively. By the removal of an Ets1 molecule, the correlations between the upstream and downstream halves of the DNA chains decreased, and those inside each half increased (a in Fig. 4). This separation of motions between the two Ets1 binding sequences indicated that the binding of dimerized Ets1 stabilizes the cooperative motions between these two DNA regions. While most of the components of Ets1 positively correlate with the first half of the DNA sequence bound to Ets1, the β-sheet (b and c in Fig. 4A) and the C-terminal loop have positive correlations with the other side of the DNA, and they correlate negatively with the other parts of the Ets1 molecule (d and e in Fig. 4). In addition, the correlations between the HI2–H1 and H2–H3 regions (f in Fig. 4), and those between the H3 helix and other regions except for the β-sheet were increased (f, g and h in Fig. 4). Since these parts (HI2–H1 loop, H2–H3 loop, and H3 helix) exhibited positive correlations with the partner Ets1 in the (Ets1)_2_–DNA model, removal of the partner resulted in the facilitation of the intramolecular correlative motions.

The differences in the contacting correlation networks between the two models are shown in Fig. S11A. As shown in the heat map (Fig. 4), the Ets1–DNA contacting pairs correlate more positively than those in the (Ets1)_2_–DNA model (the green, lime, orange, and gray arrows in Fig. S11A). For the intramolecular interactions in Ets1, the two loops HI2–H1 and H2–H3 contacted at Ile335–Trp375, and the distances did not significantly change during both simulations, the single Ets1-DNA and (Ets1)_2_–DNA (Fig. S11C). However, the mDCC value at Ile335A–Trp375A was larger in the single Ets1-DNA than that in the (Ets1)_2_–DNA (0.772 and 0.613 in the single Ets1–DNA model and the (Ets1)_2_–DNA model, respectively, as shown by the black arrows in Fig. S11A and B).

### Effect of the N380A Mutation on the Correlation Network

The results of the MD simulations in the (Ets1)_2_–DNA model discussed above emphasize the importance of Asn380, which is located at the interface of the protein–protein interactions, consistent with the previously reported mutant assay [12]. We next performed a 200 ns MD simulation for the N380A mutation model built from the crystal structure of the (Ets1)_2_–DNA complex (PDB ID: 3MFK).

The differences in the contacting correlation network from the (Ets1)_2_–DNA model are shown in Fig. S12. There were some discrepancies between the two chains of Ets1. The differences in the correlations of the Ets1–DNA interactions in the N380A model from the (Ets1)_2_–DNA model were basically greater in chain A than in chain B. The contacts at the protein–protein interface around the mutated points and their interaction partners reduced their correlations in both chains. For example, the mDCC values of Ala324A–Ala380B were 0.654 and 0.395 for the wild type and mutant, respectively. The corresponding DCC values were 0.330 and 0.105, respectively. As shown in Figs. S12B and C, the interactions between Asn380 at the H2 helix and Ala324 at the HI2 helix were disrupted and the relative positions of these helices were slightly altered. This dissociation of the interactions and the movement of the HI2 helix away from the partner H2 helix may trigger the disruption of the interplay between the two Ets1 molecules. Thus, the importance of Asn380 is supported by the current analysis for the dynamics of the Ets1 systems.

### DNA Structures

We simulated the system composed of an isolated double-stranded DNA molecule in solution, to evaluate the effects of Ets1 binding on the DNA structure (referred to as the isolated DNA model, hereafter). Consequently, the DNA structure significantly changed upon binding one or two Ets1 molecules. Ets1 binding to DNA narrowed the widths of the major grooves. The average values of the major grooves were 27.9 Å, 27.8 Å, 28.0 Å, and 28.7 Å for the (Ets1)_2_–DNA, single Ets1–DNA, N380A, and isolated DNA models, respectively. The details are shown in Fig. S13A. Since the recognition helix H3 is embedded in the major groove, the tighter major groove in a complex structure must be preferred for the recognition of H3.

Next, we assessed the conformational changes at C110, which intersected the correlation pathways by contacting the H1, H2, and H3 helices (orange, lime, and green arrows shown in Fig. 2), as discussed above. The structural parameters of DNA, defined by 3DNA software [25], were computed for the significant conformational changes in the single Ets1–DNA, N380A, and isolated DNA models from the (Ets1)_2_–DNA model. The gains in the ensemble averages of the geometrical descriptors “Slide” and “X-displacement”, which are defined as the displacements of a base pair along the plane orthogonal to the helix axis in 3DNA, from the isolated DNA to the single Ets1–DNA were 0.328 Å and 0.53 Å, respectively. Those from the isolated DNA to the (Ets1)_2_–DNA were 0.975 Å and 1.61 Å, respectively. The details are shown in Fig. S13B and C. These displacements of the base pairs can be interpreted as the result of interactions with the Tyr395A side-chain, the Tyr396A backbone, (the green arrow) and the Leu337A backbone (the orange arrow), and the homo-dimerization of Ets1 affects these interactions and facilitates this conformational change at C110.

### Summary

**Figure 5:**
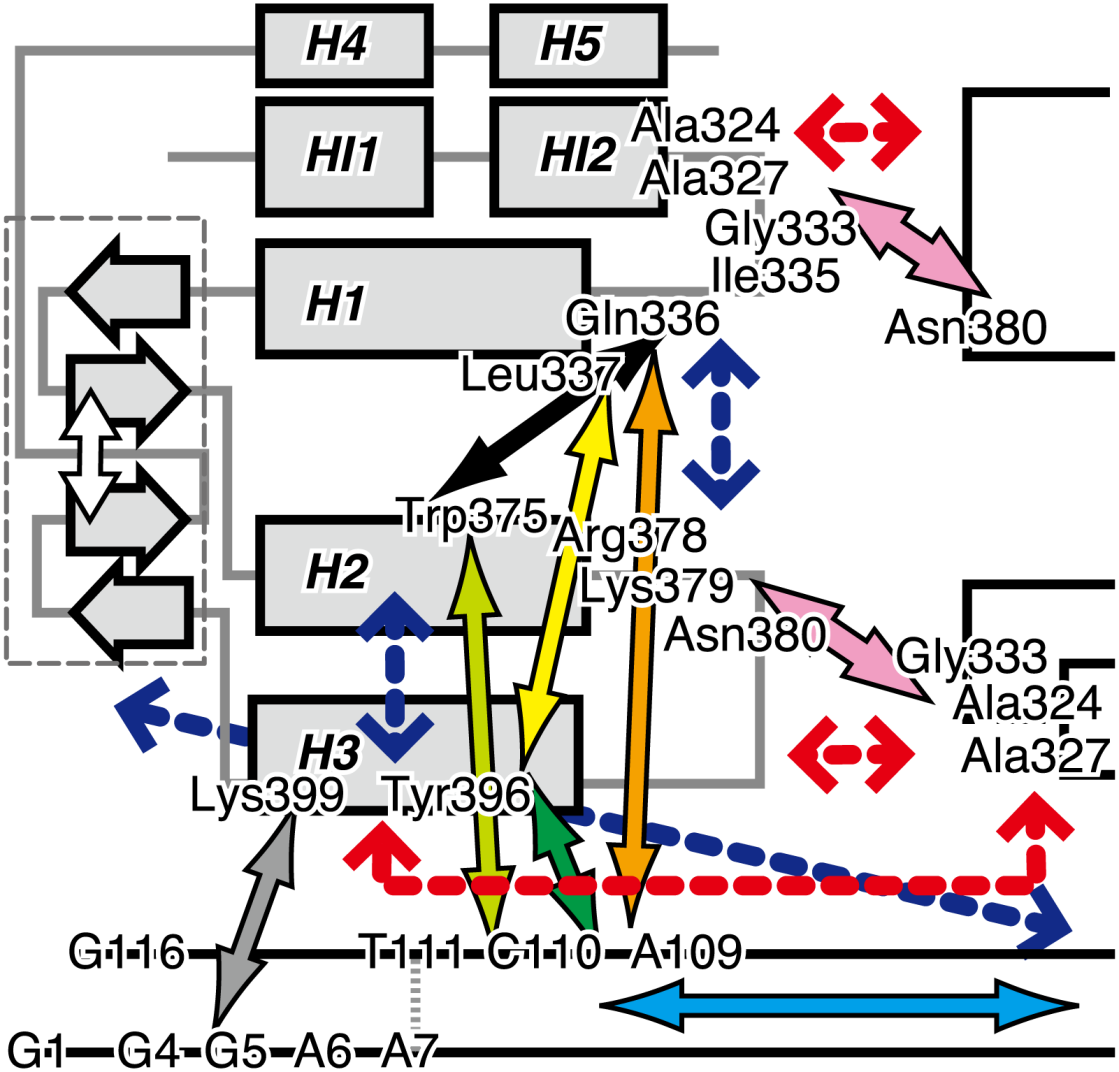
Summary of the correlation network in the (Ets1)_2_–DNA complex. The colored bold arrows indicate interactions of important residues that exhibited high Betweenness values. The colors of these arrows are consistent with those in the other figures. The red dashed arrows indicate the intermolecular pairs with highly positive correlations between the Ets1 molecules. The blue dashed arrows indicate the pairs with significant gains of correlations by the loss of the partner Ets1 molecule.

We developed the multi-modal extension of the DCC method, named mDCC, which can be interpreted as the decomposition of the DCC value into each pair of modes of motions. We applied it to the analysis of Ets1 dimerization upon binding to the Stromelysin-1 gene promoter. Fig. 5 summarizes the correlation network in this molecular assembly analyzed in this study. The homo-dimerization of Ets1 on this palindromic regulatory element modifies the motions of the recognition helix (H3) to correlate with the partner Ets1, while they did not directly contact. The results of the mDCC analysis suggest that the effects from the partner are propagated via two pathways: (i) direct protein–protein interactions at the HI2–H1 and H2–H3 loops, such as the hydrogen bond between the Asn380 side-chain and the Gly333 backbone (the pink arrows), and (ii) the pathway through DNA (the cyan arrow). These two pathways are interconnected by direct contacts among the DNA (A109, C110, and T111), the N-terminus of the H1 helix (Ile335, Gln336, Leu337, Asn380) and the H3 helix (Tyr395, Tyr396, and Lys399), and the C-terminus of the H2 helix (Trp375, Arg378, and Lys379) shown as bold arrows in different colors (The details are provided in the legend for Fig. 5). While the two loops contacting the partner Ets1 correlate positively with the partner, rather than with the regions inside the same molecule, the artificial removal of the partner increases the correlations of the intramolecular pairs of these regions. These loops and the H3 helix switch their correlating partner from inside the Ets1 to the neighboring Ets1 by homo-dimerization. In addition, the strongly negative correlations of the β-sheet with the other regions inside the same molecule are moderated by homo-dimerization, which could stabilize the entire structure of Ets1. Furthermore, our mDCC analysis revealed that the important intermolecular contacts are transiently switched by side-chain flipping (Fig. 2).

The Betweenness analysis on the correlation networks quantifies importance of each residue for the intermolecular communications, and some high Betweenness residues agreed with the precedent mutation studies, *e.g.*, Asn380 and Gly333 [12]. It suggests the applicability of our method to a prediction of new target residues for mutation experiments.

## Materials and Methods

We constructed four molecular models originating from the crystal structure of the (Ets1)_2_–DNA complex (PDB-ID: 3MFK): (i) the (Ets1)_2_–DNA model, composed of the same components as the crystal structure, (ii) the single Ets1–DNA model, prepared by the removal of an Ets1 molecule (chain B) from the crystal structure, (iii) the N380A mutation model, prepared by introducing the N380A mutation to the two Ets1 molecules in the crystal structure, and (iv) the isolated DNA model, made by removing the two Ets1 molecules from the crystal structure. All models were bathed in a 150 mM NaCl solution in a periodic boundary cube with at least 15 Å margins from the circumscribed box of the solute, in all six directions. Equilibrations of the systems were done with the GROMACS software [26], according to the following procedure. The steepest descent and the conjugate gradient methods for energy minimization were performed first. Then, a MD simulation in the NPT ensemble (Berendsen barostat) with position restraints on the heavy atoms was performed during 1.0 ns, in which the first 500 ps involved gradual heating of the system from 10 K to 300 K, and 300 K was maintained for the succeeding 500 ps, using 0.5 fs time steps. Finally, the systems were equilibrated by the NPT ensemble for 5.0 ns without any position restraint, but with the LINCS constraint [27], using 1.0 fs time steps. Electrostatic potentials in the equilibrium runs were calculated by the particle mesh Ewald (PME) method [28]. The production runs, with the initial structure that was the final one for the equilibrium simulation, were performed by the myPresto/psygene-G software [29], which is our original MD simulation program specialized for GPGPU computation. We applied the zero-dipole summation (ZD) method for the computation of electrostatic potentials without the Fourier space calculations [30]. This method has been extensively evaluated for several molecular systems, including proteins and DNA, and it has been confirmed that the energy errors of the ZD from the PME were quite small [31]–[33]. In all simulations, the AMBER99SB force field [34] with the bsc0 correction [35] and the ion parameters presented by Joung and Cheatham [36] were applied for bonded and non-bonded potential energy calculations. For each of the four models, the simulation was performed during 200 ns while keeping the system temperature at 300 K by the Hoover-Evans thermostat, with a 1.0 fs time step. The first 10 ns of the trajectories were not used in the correlation analyses. In addition, 100 ns simulation on (Ets1)_2_–DNA model was performed with different initial atomic velocities for checking the robustness of results.

mDCC analyses were performed with our in-house software, which is a modified version of the program used in our previous studies [37], [38]. The structural parameters for DNA were calculated by using the 3DNA software [25]. Other in-house scripts for data handling and analyses were powered by MDAnalysis [39]. The figures of the 3D structures of molecular systems were drawn with VMD [40] and Pymol [41]. The 2D networks were visualized by Cytoscape [42].

## Supporting Information

Figure S1
**Schematic illustration of the differences between the conventional DCC (A) and our new approach, named mDCC (B), for a simple toy-model.** (A) Images of the trajectories of the *i*th and *j*th particles are shown as gray polygonal lines on the left and right sides of the figure, respectively. The magenta crosses indicate averaged coordinates of each particle, which are the bases of the calculations of fluctuation (*<r_i_(t)>_t_*). In this figure, the four time points, *t = t_1_*, *t_2_*, *t_3_*, and *t_4_*, are highlighted by arrows representing the displacement of the *i*th particle from its average at *t* (*Δr_i_(t)*). The DCC value should be close to zero for this pair of particles, because the *i*th particle drifted along the vertical axis, but the *j*th one fluctuated along the horizontal axis. However, these particles correlatively vibrated along the horizontal direction. The conventional DCC approach cannot find such correlations of rapid fluctuations hidden in a large drifting motion. (B) Our new approach, mDCC, tackles this problem by considering multi-modal distributions of coordinates. A pattern recognition technique is applied to find modes of motions as the Gaussian mixture distributions. The centers of two modes of the *i*th particle marked with the magenta crosses (here we refer to them as 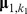 and 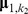), and the displacements from these two centers at the time *t* are shown as 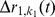 and 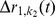. A mDCC value is defined for each pair of modes; *i.e.*, correlations for pairs of *k_1_–l_1_* and *k_2_–l_1_* are calculated in this case. At *t = t_1_* or *t = t_2_*, the first particle is likely to be assigned to the mode *k_1_*, because the particle is located near the center of the mode *k_1_* (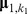). The contributions from these snapshots are more heavily considered for the calculation of the *k_1_–l_1_* correlation than that of *k_2_–l_1_*. Precisely, the weighted values were decided in terms of the ratio of probability density functions, as Eqs. (5) and (6) in the main text. In the same way, deviations from 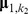 were significantly considered at later times (*t = t_3_, t_4_*). As a result, this approach characterizes the motion of the *i*th atom as fluctuations along the horizontal axis centered at 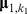 and 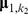 for the first and last half of the trajectory, respectively. The mDCC approach can find correlating small fluctuations during non-correlating large drifting motions. (C) The spatial distributions of the particle coordinates in the simulation on the toy-model. Cyan balls indicate positions of particles in each step, red meshes are contours of inferred Gaussian functions, and yellow balls are their centers. The two particles stably fluctuated along the x-axis, based on 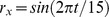
_,_ during the entire trajectory (1,000 steps), but the first particle linearly drifted along the y-axis from steps 480 to 520. The pattern recognition technique defined two Gaussian functions that correspond to the first (mode *k_1_*) and last (mode *k_2_*) half of the trajectory, in order to model the spatial distribution of the first particle, respectively. The motion of the other particle was recognized as a uni-modal distribution (mode *l_1_*). As a result, the mDCC values for both pairs *k_1_–l_1_* and *k_2_–l_1_* showed highly positive correlations (*mDCC(1,2; k_1_, l_1_)* = 0.968, and *mDCC(1,2; k_2_, l_1_)* = 0.968), although the conventional DCC method could not find such a high correlation (*DCC(1,2)* = 0.582). In the mDCC calculation, the trajectory was divided into the two parts: a time range when the first and second particles belong to modes *k_1_* and *l_1_*, and that when they belong to modes *k_2_* and *l_1_*, by the weighting coefficient 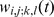. In addition to the correlation coefficients, we can assess the probability of each pair, which were 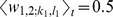 and 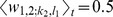. (D) An illustration of the parameters for the mDCC calculation in the toy-model simulation. For the first particle, the assignment to the Gaussian functions (*k_1_* or *k_2_*) at each step was determined by the y-axis coordinates, and the particle was assigned to mode *k_1_* and *k_2_* in the first and last half of the trajectory, respectively (the left-top and right-top plots). On the other hand, the second particle stayed at the same y-position and was always assigned to mode *l_1_* (the left-bottom plot), which means *p_l1_(r_2_)* is always 1.0. *mDCC(1,2; k_1_, l_1_)* was calculated by using trajectories with non-zero 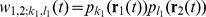 (the right-bottom plot, and the area colored as pink in the left plots). The last half of the trajectory (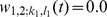) was ignored for the calculation of *mDCC(1,2; k_1_, l_1_)*.(EPS)

Figure S2 **Statistics of 6,886 Gaussian elements inferred for 2,887 heavy atoms in the (Ets1)_2_–DNA model.** The Gaussian elements fell into five categories, in terms of the number of Gaussian elements in the same atom, from one to five. The statistics was determined for each category and they are colored green, blue, red, cyan, and purple for the ascending order of numbers of elements. For example, the statistics of Gaussian elements, each belonging to an atom with five Gaussian elements, are shown in purple. (A) The histogram of Gaussian elements against π*_k_*. (B) The number of atoms with a certain number of Gaussian elements. (C) The boxplot of RMSF of atoms. The black dots are RMSF values beyond the standard deviations.
(EPS)

Figure S3 **Evaluations of effects of the parameter** ***K*** **and choice of representative atomic pairs for each residue pair, using the (Ets1)_2_–DNA model.** (A) The mDCC map calculated in the condition *K* = 10 (the upper triangle) and its differences from that in *K* = 5 (the lower triangle). (B) The mDCC map of pairs of Cα and C5’ atoms for amino acid and nucleotide residues, respectively. The upper and lower triangles indicate the values of mDCC and its differences from the original mDCC map, which is calculated for the atomic pairs with the maximum mDCC values in each residue pair (Fig. 1A), respectively.
(EPS)

Figure S4 **Evaluations of the robustness of the mDCC analysis on (Ets1)_2_–DNA model.** These mDCC maps calculated by the different conditions. The upper and lower triangles indicate the mDCC values and their differences from the original mDCC map shown in Fig. 1A, respectively. (A, B) The results of analyses on some different time ranges of trajectories. The time range from 10 to 100 ns and that from 110 to 200 ns, for the panels (A) and (B) respectively. (C) The mDCC map calculated from the alternative run for the time range from 10 ns to 100 ns with a different set of initial velocities. (D) The mDCC map calculated by using another random variable defining the initial guess for the pattern recognition (denoted as mDCC’).
(EPS)

Figure S5 **The time evolution of the probability density function of each Gaussian element in the Gaussian mixture distribution for the Nη atom of Asp380B, in the trajectory of the (Ets1)_2_–DNA model.** In this atom, four Gaussian elements with π*_k_* = 0.683, 0.175, 0.103, and 0.039 were defined, and their *pdf(r_i_)* are plotted in cyan, pink, purple, and yellow dots (the yellow plots are hidden in the background of the other plots because of very low probabilities), respectively.
(EPS)

Figure S6 **An example explaining Betweenness in a graph.** This measure quantifies the centrality of each node and a node at the center of the graph should exhibit high Betweenness. Nodes colored magenta and cyan exhibit high and low Betweenness values, respectively. In this example, the node bridging the two cliques shows the highest Betweenness, because all of the shortest paths between a node in the left clique and that in the right one include the bridging node. On the other hand, nodes at distal positions show low Betweenness since there are no shortest paths through them.
(EPS)

Figure S7 **The correlation network shown in** **Fig. 3A** **.** The three circles correspond to the Ets1 molecule of chain A (the left circle), that of chain B (the right circle), and the double-stranded DNA (the center circle). Each node means a residue with one character indicating the type of amino acid and nucleotide (asterisks mean the N- and C-terminal caps of peptides). They are ordered by the sequence along the counter-clockwise direction, beginning at the bottom, and the black filled circles adjoined the nodes are drawn for every ten residues (Asp310, Val320, Thr330, …, His430). Nodes with higher Betweenness values are filled by darker colors. The border colors of them mean the secondary structure: green, orange, and cyan mean the α-helix, β-sheet, and others. The edges are drawn between nodes with the contacts (<5.0 Å) and positive correlations (mDCC ≥0.5), and the color gradation of edges indicates mDCC values from 0.5 to 1.0. The edges shown as zigzag lines indicate transiently correlated residue pairs (DCC<0.5). The bold arrows indicate some important interactions discussed in the main text.
(EPS)

Figure S8 **Interactions around some high Betweenness residues, in the (Ets1)_2_–DNA model.** (A, B, and C) The interactions among Arg378A, Ala324A, and Glu343A. (A) A snap shot around the interacting residues. Arg378A, Ala324A, Glu343A and the water molecule mediating Arg378A–Ala324A interaction are shown as sticks. The dashed lines indicate the atom pairs analyzed in the panels (B) and (C). (B) Time evolution of the distances from the Nη2 atom of Arg378A to the Oε1 and Oε2 atoms of Glu343A (the cyan and pink plots, respectively). (C) Time evolution of the distance between the Nη2 atom of Arg378A and N atom of Ala324A. (D and E) The interaction between Trp375A and T111. (D) A snap shot around the interacting pair. (E) Tme evolution of the distance between the Nε1 atom of Trp375A and OP2 atom of T111.
(EPS)

Figure S9 **Partial deformation of the HI2 helix in the Ets1–DNA model.** (A) A superimposed picture of 3D structures of the Ets1 molecule in the crystal structure (chain A; the green ribbon) and that in the snapshot from the MD simulation of the single Ets1–DNA model at 200 ns (the red ribbon). (B, C) Snapshots around the HI2–H1 loop in the simulation of the Ets1–DNA model at 0 ns and 150 ns, respectively. (D) Time course of the distance between the Cβ atom of Asp327A and the O atom of Gly333A, observed during the simulations of the single Ets1–DNA model (cyan) and the (Ets1)_2_–DNA model (pink).
(EPS)

Figure S10 **Comparisons of the 3D structures of Ets1 molecules between the (Ets1)_2_–DNA complex and apo-form.** (A) Structures of Ets1 molecules obtained by experimental measurements. The Ets1 molecule in the apo-form solved by the NMR study (PDB ID: 1R36, model 1) is shown as the yellow ribbon, and that in the (Ets1)_2_–DNA complex solved by X-ray spectroscopy (PDB ID: 3MFK, chain A) is shown as the red ribbon. The structures were superimposed with the coordinates of the Cα atoms in the H1 helix. Dashed circles emphasize differences in the positions of the HI2 helices. (B) A superimposed picture of Ets1 molecules at 200 ns during the MD simulation of the (Ets1)_2_–DNA model (chains A and B are shown as red and green ribbons, respectively) and the NMR structure in the, apo-form (PDB ID: 1R36, model 1; the yellow ribbon). The dashed circles highlight the HI1 helix in each structure.
(EPS)

Figure S11 **Differences between the (Ets1)_2_–DNA and single Ets1–DNA models.** (A) A network of interacting correlative pairs of residues comparing the (Ets1)_2_–DNA and single Ets1–DNA models. Nodes indicate residues, and edges were drawn between residue pairs with highly positive correlation (the maximum value of mDCC ≥0.5) and contacting (the distance between centers of modes <5 Å) in at least one of the two models. The edge colors indicate the differences in the mDCC values of the single Ets1–DNA model from that of the (Ets1)_2_–DNA model; blue edges means the pairs of residues highly correlated in the (Ets1)_2_–DNA model but not in the single Ets1–DNA model, and red edges are *vice versa*. Sizes of nodes denote the Betweenness values in the single Ets1–DNA model. The bold arrows are interactions discussed in the main text. (B) The 3D structure of the molecule at 200 ns of the (Ets1)_2_–DNA and single Ets1–DNA models. The black arrows highlight the distance between Ile335A and Lys375A. (C) The time course of the distance between the Cγ1 atom of Ile335A and the Cη2 atom of Lys375A, in the chain A of the (Ets1)_2_–DNA (cyan) and single Ets1–DNA (pink) models.
(EPS)

Figure S12 **Differences between correlation networks of the (Ets1)_2_–DNA and N380A models.** (A) A network of interacting correlative pairs of residues, comparing the results of the (Ets1)_2_–DNA and N380A models. See the legend of Fig. S11A. (B) 3D structures of the (Ets1)_2_–DNA and N380A models at 200 ns. They were superimposed based on the H2 helices. The arrows indicate the atom pairs shown in panel (C). (C) Time course of interatomic distances between the Cβ atoms of Ala324A and Asn/Ala380B in the (Ets1)_2_–DNA (the pink plot) and N380A (the green plot) models.
(EPS)

Figure S13 **Structural differences in the double-stranded DNA molecule.** (A) The widths of the major grooves. The base pair position 2 corresponds to the width between C2 and C108. The major groove recognized by the H3 helix roughly corresponds to positions 2–4 and 6–8 for chains A and B, respectively. Asterisks indicate significant differences from the (Ets1)_2_–DNA model (P-value ≤0.001, calculated by the Wilcoxon test for structures sampled every 5 ns). (B) Displacements of each base pair along the perpendicular direction against the base pair axis, defined as the “Slide” geometric parameter in the 3DNA software. The base pair position 10 corresponds to C110. (C) A snapshot of structures of the C110–G8 base pair in the (Ets1)_2_–DNA (the cyan sticks) and isolated DNA (the purple sticks) models at 200 ns. The two structures were superimposed based on T111–A7 base pair shown as thin lines. Amino acid residues interacting with C110 are also shown as cyan sticks. The bold orange and green arrows point to the interactions between the amino acid residues and C110.
(EPS)

Movie S1 **A movie for the toy-model. See the legend of Fig. S1C.**
(MPG)

Movie S2 **A movie for the 200** **ns run of the (Ets1–DNA)_2_–DNA model.**
(MPG)

Movie S3 **A movie for the 100** **ns alternative run of the (Ets1–DNA)_2_–DNA model with a different set of initial atomic velocities.**
(MPG)
